# A comparison of passive surveillance and active cluster-based surveillance for dengue fever in southern coastal Ecuador

**DOI:** 10.1186/s12889-020-09168-5

**Published:** 2020-07-06

**Authors:** Melissa Vitale, Christina D. Lupone, Aileen Kenneson-Adams, Robinson Jaramillo Ochoa, Tania Ordoñez, Efráin Beltran-Ayala, Timothy P. Endy, Paula F. Rosenbaum, Anna M. Stewart-Ibarra

**Affiliations:** 1 Institute for Global Health and Translational Science, SUNY Upstate Medical University, 505 Irving Avenue Suite 4200, Syracuse, NY USA; 2 Department of Public Health and Preventive Medicine, SUNY Upstate Medical University, 750 East Adams Street, Syracuse, NY USA; 3 College of Medicine, MD Program, SUNY Upstate Medical University, 750 East Adams Street, Syracuse, NY USA; 4 Ministry of Health, Machala, El Oro Ecuador; 5 Department of Medicine, Universidad Técnica de Machala, Machala, El Oro Ecuador; 6 Department of Microbiology and Immunology, SUNY Upstate Medical University, 750 East Adams Street, Syracuse, NY USA; 7 Department of Medicine, SUNY Upstate Medical University, 750 East Adams Street, Syracuse, NY USA; 8 Department of Montevideo, Inter-American Institute for Global Change Research, Montevideo, Uruguay

**Keywords:** Dengue, Active surveillance, Passive surveillance, Latin America, Arboviral, Public health intervention, Ecuador

## Abstract

**Background:**

Dengue is a major emerging infectious disease, endemic throughout the tropics and subtropics, with approximately 2.5 billion people at risk globally. Active (AS) and passive surveillance (PS), when combined, can improve our understanding of dengue’s complex disease dynamics to guide effective, targeted public health interventions. The objective of this study was to compare findings from the Ministry of Health (MoH) PS to a prospective AS arbovirus research study in Machala, Ecuador in 2014 and 2015.

**Methods:**

Dengue cases in the PS system were compared to laboratory confirmed acute dengue illness cases that entered the AS study during the study period. Variables of interest included age class and sex. Outbreak detection curves by epidemiologic week, overall cumulative incidence and age-specific incidence proportions were calculated. Descriptive statistics were tabulated for all variables of interest. Chi-square tests were performed to compare demographic characteristics between the AS and PS data sets in 2014 and 2015.

**Results:**

177 and 245 cases were identified from 1/1/2014 to 12/31/2015 by PS and AS, respectively; nine cases appeared in both systems. AS identified a greater number of laboratory-confirmed cases in 2014, accounting for more than 60% of dengue cases in the study area. In 2015, the opposite trend was observed with PS identifying 60% of the dengue cases in the study area. Peak transmission time in laboratory confirmed dengue illness, as noted by AS and PS was similar in 2014, whereas earlier detection (7 weeks) was observed by AS in 2015. Younger patients were more frequently identified by PS, while older patients were identified more frequently by AS. The cumulative incidence proportion for laboratory confirmed dengue illness reported via PS to the MoH was 4.12 cases per 10,000 residents in 2014, and 2.21 cases per 10,000 residents in 2015.

**Conclusions:**

Each surveillance system captured distinct demographic subgroups within the Machala population, possibly due to differences in healthcare seeking behaviors, access to care, emerging threats of other viruses transmitted by the same mosquito vector and/or differences in clinical presentation. Integrating AS with pre-existing PS can aid in identifying additional cases in previously underdiagnosed subpopulations, improving our understanding of disease dynamics, and facilitating the implementation of timely public health interventions.

## Background

Dengue is a mosquito-borne infectious disease that causes morbidity and mortality in over 100 countries, where the disease is considered to be endemic [1]. It is the most prevalent mosquito-borne viral disease worldwide, with approximately 2.5 billion people at risk for the disease and 400 million people infected annually [2]. The rapid growth of urban areas throughout the dengue endemic regions of the world has increased the number of people at risk of disease [3]. Since the 1980's, dengue has rapidly reemerged in the Americas, increasing in incidence, severity, and geographic distribution. The four dengue virus serotypes (DENV1–4) co-circulate in endemic regions [4] and are transmitted primarily by the female *Aedes aegypti* mosquito. Dengue is a complex disease that is influenced by a combination of social determinants, vector populations, public health interventions, land use and vegetation, and climate across timescales [5, 6].

The clinical manifestations of dengue can vary widely. Disease can range from subclinical (asymptomatic), to mild febrile illness, to more severe flu-like illness, and in fewer cases, to shock and/or death. There are currently no targeted therapeutic treatments available in most parts of the world beyond supportive care and close observation. Access to the only licensed dengue vaccine (Sanofi’s Dengvaxia©) is also limited. The vaccine is currently recommended only for use in dengue-seropositive individuals due to long term safety issues observed in seronegative individuals in the safety follow-up trials [7, 8].

The primary aims of dengue surveillance are rapid detection of epidemics for early interventions, to assess the burden of disease across subpopulations, to monitor spatiotemporal trends in disease distribution, and to evaluate and plan interventions [9, 10]. Both active and passive surveillance methods are utilized in tracking dengue infections worldwide. Active surveillance (AS) is a resource intensive approach whereby members of the community are tested for dengue regardless of symptom status [10]. Passive surveillance (PS), a less resource-intensive approach, is the accepted standard for dengue surveillance in many countries with mandatory reporting of dengue cases [11]. Passive surveillance accounts for those who recognize that they are sick and choose to seek treatment in a clinical setting. Anyone who does not seek treatment is not counted in PS, resulting in underreporting of disease cases [11].

The Global Strategy for Dengue Prevention and Control highlights the importance of combining epidemiological information from targeted, local AS studies with broader PS systems to improve dengue control [10]. AS studies often capture significantly more cases of dengue, and an earlier peak in cases, than are reported via PS [12–16]. One study found that AS identified a 10-fold higher dengue case load as compared to the national PS systems in Latin America (Brazil, Columbia, Mexico) [12]. A study in Nicaragua reported 21 times more cases via AS per year as compared to the PS system [13]. In French Guiana, AS was able to detect a dengue outbreak 3 to 4 weeks earlier than PS [17]. Despite research demonstrating that AS is a sensitive tool for estimating disease burden, AS is rarely implemented as an operational public health approach, due in large part to the expense and logistics required [2, 6, 11]. For this reason, AS data from sentinel sites and research studies around the world provide key insights into dengue dynamics.

The objective of this study was to compare dengue illness/infection data reported to the Ministry of Health (MoH) PS system to cases detected via a prospective AS arbovirus study in a dengue endemic region of Ecuador. Dengue is a mandatory notifiable disease in Ecuador. We compared the incidence of disease, the demographic profile of cases, and timing of the case reports over 2 years. We hypothesized that the AS would detect a greater incidence of disease, earlier peaks in annual transmission, and more infections in demographic subgroups prone to underreporting.

## Methods

### Study site

All data used for this comparative analysis were collected from Machala, Ecuador, a coastal port city in southern Ecuador and the capital of El Oro province; there were approximately 270,000 residents at the time of this study (2014–2015). The incidence of dengue and the density of *Ae. aegypti* mosquitoes in Machala is amongst the highest in Ecuador, as well as other Latin American countries and Asia [18–21]. Dengue is transmitted seasonally, with more cases reported during the hot, rainy season from February to May. Dengue outbreaks have been observed to correlate with extreme climate events, such as El Niño, that strongly impact local rainfall and temperatures in southern coastal Ecuador [5, 18, 22].

We selected four (of 23) sentinel outpatient clinics located around Machala and operated by the MoH; sites were chosen based on a high burden of dengue in the community catchment areas and their interest and ability to participate in the study [19]. The clinics included Brisas del Mar, Rayito de Luz, Mabel Estupiñan, and El Paraiso. In addition, the Teófilo Dávila Hospital, the primary public hospital run by the MoH, was included as it is the province-level reference hospital where the outpatient clinics refer patients with severe dengue illness [19]. Public clinics and hospitals are required to report cases of dengue-like illness (with and without warning signs) to the MoH for patients seeking care.

### Active surveillance

**Fig. 1:**
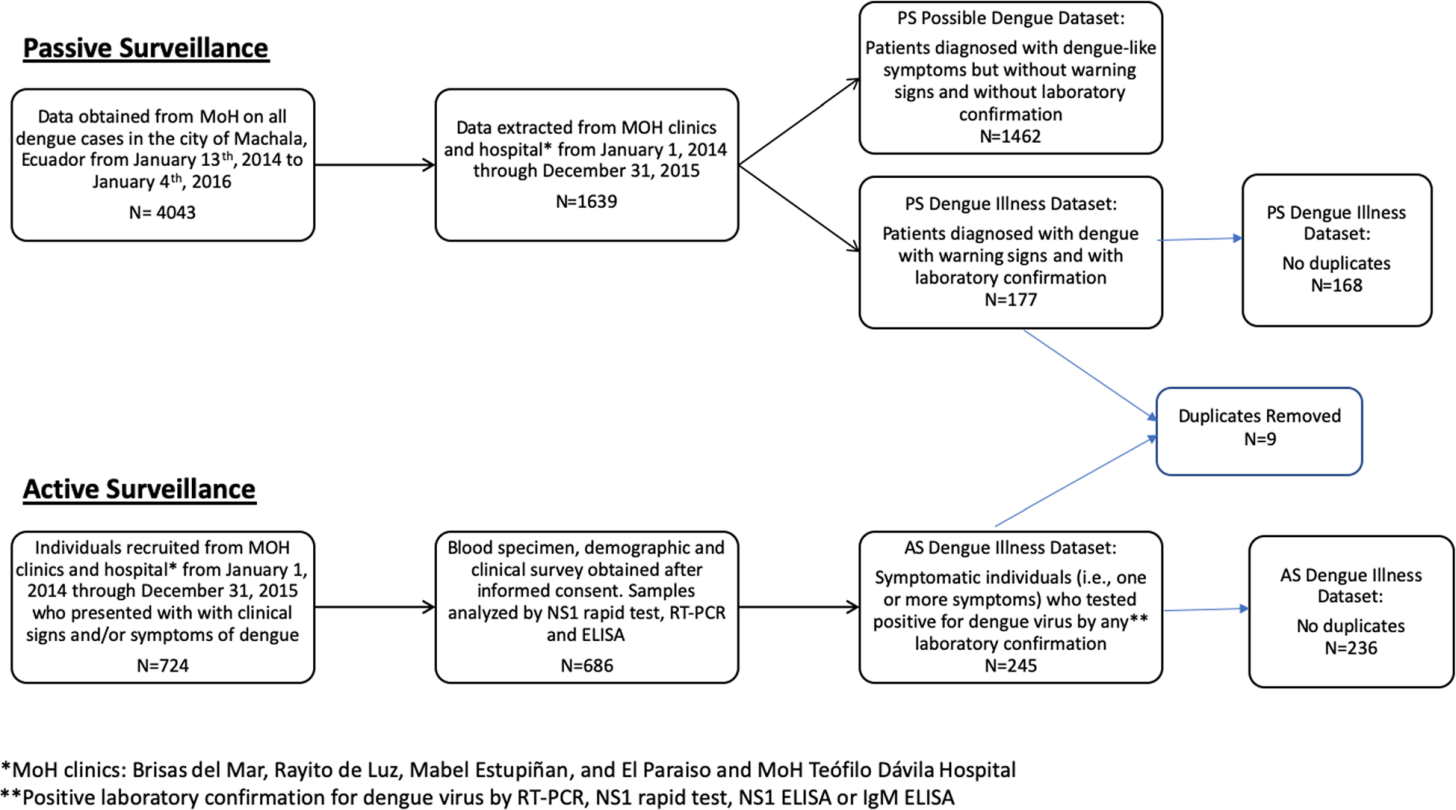
Flowchart of active and passive surveillance recruitment methods. Epidemiological information on dengue cases was obtained from an active surveillance (AS) research study and passive surveillance (PS) operated by the Ministry of Health in the dengue-endemic city of Machala, Ecuador, in 2014 and 2015. The diagram indicates the process by which data were obtained, the inclusion criteria, and duplicates from systems (*n* = 9) that were excluded

Figure 1 provides a flowchart of AS and PS recruitment methods for the study. The AS study design and diagnostic procedure have been described previously [19]. Briefly, individuals (index subjects) were recruited into the AS research study after visiting one of the four MoH clinics or the Teofilo Davila Hospital with clinical signs and/or symptoms of dengue (see Fig. 1). Index subjects were referred to our study technician or nurse; informed consent obtained, and demographic and clinical information recorded. At the time of clinical evaluation, a 20 ml blood specimen (adjusted for age and weight by United States National Institutes of Health criteria) was obtained by venipuncture from each participant. Samples were processed at the diagnostic laboratory within the Teofilo Davila Hospital. Acute dengue infections were confirmed via blood serum with NS1 rapid strip tests. A maximum of four index subjects that tested positive for dengue were randomly selected each week to participate in the community surveillance component of the study. Members of the index subject’s household and members of four neighboring households within a 200-m radius of the index household, the typical flight range of the *Ae. aegypti* mosquito, were invited to participate in the study. The same demographic and clinical information were gathered from these individuals, as well as a blood sample.

Blood specimens were separated via centrifuge into serum, cells, and plasma and stored at − 80 °C. Samples were tested for dengue using NS1 and IgM enzyme-linked immunosorbent assay (ELISA) at the laboratory in Machala. Samples were then shipped to SUNY Upstate Medical University where reverse transcriptase polymerase chain reaction (RT-PCR) was used to confirm dengue infections and virus serotypes [19]. Positive cases of dengue in the AS system (herein called dengue illness) were defined as individuals with the presence of one or more of the following symptoms: fever, nausea/vomiting, rash, muscle/joint pain, abdominal tenderness, bleeding, diarrhea, headache, retro-orbital pain, drowsiness/lethargy, who tested positive for dengue virus by RT-PCR, NS1 rapid test, NS1 ELISA or IgM ELISA. There were 33 individuals in 2014 and six in 2015 in the AS system who had positive laboratory tests but no symptoms. These cases were excluded from the analyses reported in this manuscript since they did not fit the definition of dengue illness (symptoms and positive lab confirmation).

### Passive surveillance

Ecuador has a mandatory PS reporting strategy for dengue with and without warning signs as well as for other mandatory reportable health conditions. According to the World Health Organization (WHO) dengue guidelines [1], dengue without warning signs is defined as fever plus any two of following symptoms: nausea/vomiting, rash, aches/pains, positive tourniquet test, and leukopenia. Dengue with warning signs includes the definition above for dengue without warning signs and at least one of following warning signs: abdominal pain/tenderness, persistent vomiting, clinical fluid accumulation, mucosal bleed, lethargy, restlessness, liver enlargement > 2 cm, and/or increased hematocrit concurrent with rapid decrease in platelets [1]. If the patient is classified as having dengue with warning signs, they are admitted to the local hospital, a physical exam is administered, and a blood serum sample collected. The sample is used to confirm dengue infection via RT-PCR at the national reference laboratory of the MoH in the neighboring city of Guayaquil. Patient demographics (age, sex, pregnancy status if applicable), clinical characteristics (start date of symptoms, final clinical diagnosis), and diagnostic laboratory results are recorded. If dengue diagnosis is ruled out, an ‘other’ diagnosis is recorded. If the patient seeking care is classified as dengue without warning signs, their information is entered into a separate MoH PS dataset based on clinical symptoms and not via RT-PCR confirmation. Patients classified as dengue without warning signs are entered into the database in ‘group form’ by reporting institution or clinic, but without names or specific ages.

For this analysis, the MoH provided de-identified data on reported dengue cases from the entire city of Machala to the study team. We created two PS datasets by extracting dengue cases from these MoH reports for the same sentinel clinics/hospital utilized in the AS study from January 1, 2014 through December 31, 2015. In the primary dataset, we identified patients diagnosed with dengue with warning signs and with laboratory confirmation (referred to as dengue illness). In the second data set, we included cases with dengue-like symptoms but without warning signs and without laboratory confirmation (referred to as possible dengue).

Dengue cases in the two PS datasets were compared to dengue illness cases that entered the AS study during the same period of time. Duplicate patients were identified by matching the date, sex, and age. Variables of interest included age class (< 5, 5–19, 20–64, 65+), and sex (male, female), and pregnancy status (pregnant, not pregnant). The pregnancy status variable was not available in the PS data set without warning signs. Due to the small sample size, we were not able to compare pregnancy status across surveillance systems.

### Statistical methods

Microsoft Excel (Version 16, Microsoft Corporation, Redmond, Wash, USA) was used for data quality assessment. The Statistical Package for the Social Sciences (SPSS - IBM Corp. Released 2013, IBM SPSS Statistics for Windows, Versions 25 and 26, Armonk, NY: IBM Corp.) was used to calculate overall cumulative incidence and age specific incidence proportions, to construct outbreak detection curves by epidemiologic week, and to run all analyses. Descriptive statistics were tabulated for all variables of interest. As the demographic variables were categorical, chi-square tests were performed to compare cohort characteristics between the AS and two PS data sets in 2014 and 2015. The initial comparisons were between AS and PS dengue illness, while a second set of analyses evaluated AS dengue illness, PS dengue illness and PS possible dengue across demographics. Duplicate study subjects (*n* = 9) were omitted from both the PS and AS systems in these chi-square analyses. Results with a *p* value of ≤0.05 were considered statistically significant. Results were not corrected for multiple comparisons.

Cumulative incidence proportions (overall and age-specific) were calculated for cases identified by the MoH PS system (dengue illness and possible dengue) using the city of Machala population estimate for 2016 (*n* = 276, 691) and the age-specific population figures for Machala, also for that year [23]. Note that the hospital serves the entire population of Machala and the four sentinel clinics serve a subset of the city’s population. However, incidence calculations are based on the entire population of the city of Machala, to provide a common denominator. Cumulative incidence proportions displayed are per 10,000 persons per year.

## Results

Overall, more cases of dengue illness were identified in 2014 than in 2015. The AS study identified 205 cases of dengue illness, accounting for approximately 60% of disease burden that year in the study catchment area, while PS identified 116 cases of dengue illness in 2014, with four duplicate cases observed. In 2015, AS identified 40 dengue illness cases while PS identified 61 cases of dengue illness, with five duplicate cases. Accordingly, the overall incidence proportions for dengue illness identified by PS was greater in 2014 (4.12 cases per 10,000 people) than in 2015 (2.21 per 10,000 people, Table 1). As noted earlier, the MoH PS also registered cases of possible dengue (clinical symptoms only, but without warning signs and laboratory confirmation). In contrast to the cases of dengue illness, fewer cases of possible dengue were reported in 2014 (*n* = 650, incidence 23.49 cases per 10,000 people) than in 2015 (*n* = 812, incidence 29.34 cases per 10,000 people) (Table 1). Of note, while AS and PS were conducted in parallel, we identified only nine cases that appeared in both systems over the study period (Fig. 1). Cumulative incidence of dengue, combining both dengue illness and possible dengue cases from PS, were similar at 27.6 per 10,000 residents in 2014 and 31.55 per 10,000 residents in 2015.

**Table 1:** Dengue incidence by age from Passive Surveillance (PS)

	PS Dengue Illness	PS Possible Dengue
**2014**	***N*** **= 116**	***N*** **= 650**
Overall	4.12	23.49
Age Groups		
0–4	1.86	17.11
5–19	9.44	39.72
20–64	2.26	17.58
65+	0.54	10.21
**2015**	***N*** **= 61**	***N*** **= 812**
Overall	2.21	29.34
Age Groups		
0–4	2.23	15.25
5–19	4.10	34.14
20–64	1.39	29.80
65+	0.54	25.26

Overall cumulative dengue incidence and incidence by age classes were compared for passive surveillance (PS) dengue illness (defined as symptoms, warning signs and laboratory confirmation) and PS possible dengue (defined as clinical symptoms only, without warning signs and without laboratory confirmation) per 10,000 residents in Machala per year. Cases included in the incidence calculations are from sentinel study sites only

We calculated the incidence rates by age class for PS cases and found that children aged 5–19 had the highest incidence rates across PS datasets in both years, almost double the overall incidence rates (Table 1). Older adults (65 years or older) had the lowest incidence rates, with the exception of the possible dengue cases in 2015, where infants (0–4 years) had the lowest rates (Table 1).

We compared the demographics (sex and age classes) of dengue cases identified through the three datasets: (1) AS with symptoms and lab confirmation (dengue illness), (2) PS with warning sign symptoms and lab confirmation (dengue illness), and (3) PS with symptoms but no warning signs or lab confirmation (possible dengue). In 2014 and 2015, there were no significant differences in the frequencies of males and females with dengue identified via AS versus the two PS data sets; however, females made up a higher overall proportion of cases in 2015 as compared to 2014 (Table 2). There were significant differences in the proportion of dengue cases by age group amongst the three systems in 2014 (*p* = 0.004) and 2015 (*p* < 0.001, Table 2). In 2014, children 10–14 years of age had the greatest proportion of dengue illness PS cases (39.3%), whereas adults 20–64 years of age had the greatest proportion of AS cases (45.9%) and possible dengue PS cases (40.8%). In 2015, children aged 5–9 comprised the greatest proportion of dengue illness PS cases (38.4%), a younger age group than in 2014. Adults 20–64 years of age had the greatest proportion of AS cases (47.5%) and possible dengue PS cases (55.3%) in 2015, as was also observed in 2014.

**Table 2:** Comparison of demographics of dengue cases in 2014 and 2015

		AS Dengue Illness (DI)		PSDI		ASDI vs PSDI	PS Possible Dengue (PD)		ASDI vs PSDI vs PSPD
		N	(%)	N	(%)	***p value***	N	(%)	***p value***
**2014**									
**Sex**	**Male**	96	(47)	55	(51)	*0.70*	323	(49.5)	*0.79*
	**Female**	109	(53)	57	(49)		329	(50.5)	
**Age Groups**	**< 5**	11	(5.4)	5	(4.5)	*0.004*	46	(7.1)	*0.003*
	**5–9**	21	(10.2)	14	(12.5)		76	(11.7)	
	**10–14**	44	(21.5)	44	(39.3)		130	(20)	
	**15–19**	29	(14.1)	18	(16.1)		114	(17.5)	
	**20–64**	94	(45.9)	30	(26.8)		265	(40.8)	
	**65+**	6	(2.9)	1	(0.9)		19	(2.9)	
**2015**									
**Sex**	**Male**	16	(40)	24	(43)	*0.78*	356	(44)	*0.85*
	**Female**	24	(60)	32	(57)		448	(56)	
**Age Groups**	**< 5**	1	(2.5)	6	(7.0)	*0.11*	41	(4.4)	*< 0.001*
	**5–9**	7	(17.5)	33	(38.4)		71	(8.7)	
	**10–14**	7	(17.5)	12	(14.0)		121	(14.9)	
	**15–19**	5	(12.5)	15	(17.4)		83	(10.2)	
	**20–64**	19	(47.5)	19	(22.1)		449	(55.3)	
	**65 plus**	1	2.5)	1	(1.2)		47	(5.8)	

Active surveillance (AS) dengue illness (DI) cases were compared by sex and age groups to passive surveillance (PS) DI cases. Subsequently, sex and age groups from ASDI and PSDI were compared to PS possible dengue (PD) cases in each year of the study

**Fig. 2:**
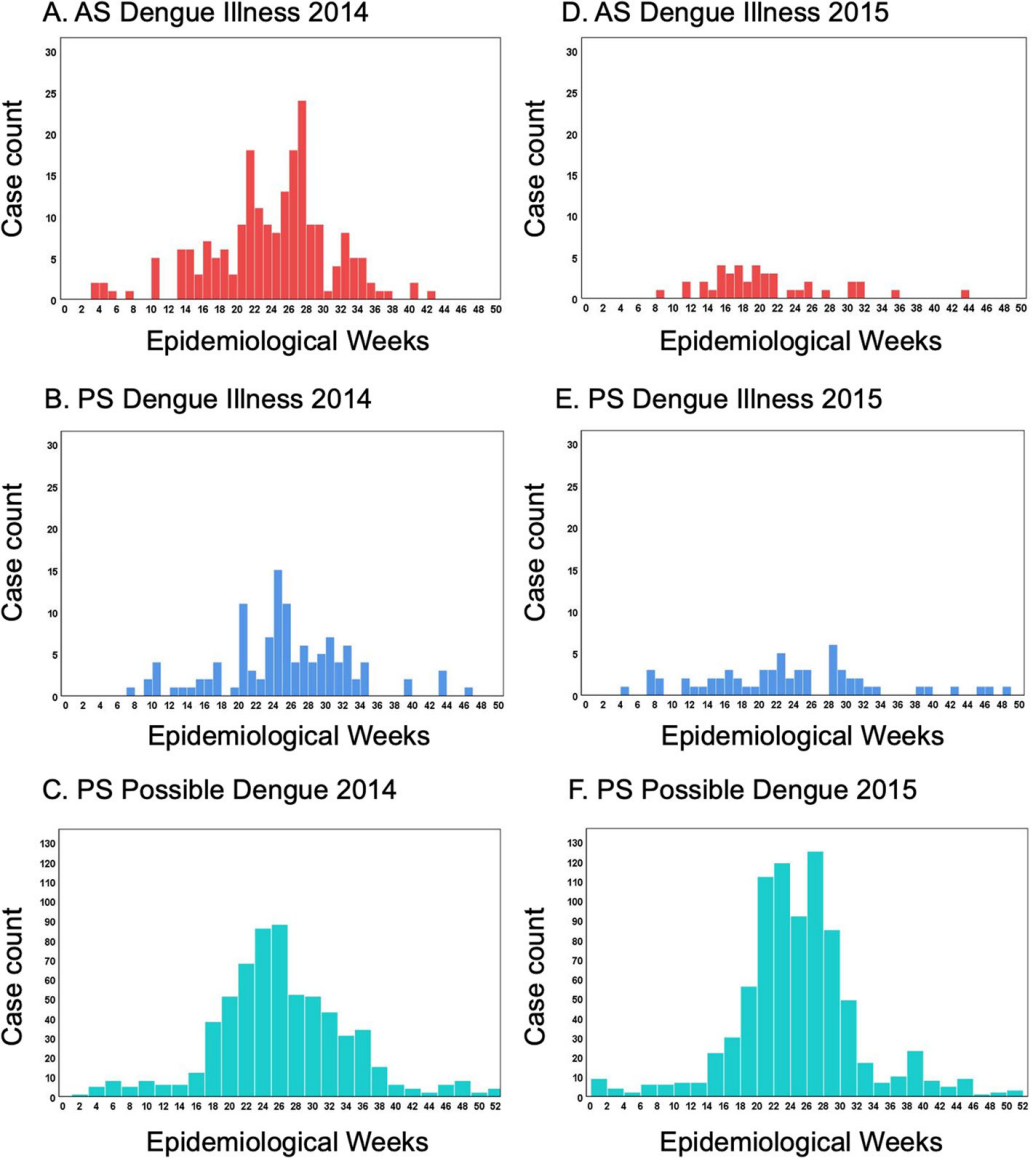
Weekly dengue cases from active and passive surveillance systems in Machala in 2014 and 2015. The number of cases of dengue illness (defined as symptomatic with positive laboratory confirmation) and possible dengue (defined as clinical symptoms only, without warning signs or laboratory confirmation) are shown by epidemiological week in 2014 and 2015 for active surveillance (AS) dengue illness (**a**,**d**), passive surveillance (PS) dengue illness (**b**,**e**), and PS possible dengue (**c**,**f**)

Epidemiological curves of both AS and PS dengue illness cases revealed bimodal transmission peaks in 2014, with the first peak for AS and PS dengue illness at 21 and 20 weeks (mid-May), respectively. The second transmission peak for PS occurred soon after, at 24 weeks (mid-June), while the second transmission peak for AS occurred later, around 27 weeks (mid-July, see Fig. 2a,b). Possible dengue cases reported via PS in 2014 peaked between 22 and 25 weeks (Fig. 2c). In 2015, there was only one transmission peak for AS dengue illness at 15–20 weeks (early April to May). PS revealed bimodal transmission peaks of dengue illness at 22 and 28 weeks (early June and mid-July, see Fig. 2d,e). Possible dengue cases in 2015 (Fig. 2f) demonstrated bimodal peak transmission at 20–24 weeks, with a slight resurgence at 26 weeks.

## Discussion

In this study, we compared the differences in demographic subgroups, timing, and incidence of dengue illness in active surveillance (AS) and passive surveillance (PS) systems in Ecuador. We found that the AS and PS captured different populations, as only nine subjects were identified as duplicates in both systems. Older individuals with confirmed dengue illness (symptoms/warning signs and lab confirmation) were more likely to be identified by AS, whereas children and adolescents with confirmed dengue illness were more likely to be reported via PS. Women made up a greater proportion of dengue cases across all surveillance systems. Although AS was able to detect an initial peak in laboratory confirmed dengue illness up to 7 weeks earlier than PS in 2015, the peak in cases was detected around the same time in 2014. Surprisingly, the proportion of total dengue cases detected by AS versus PS varied from year to year; the AS detected more dengue cases in 2014, whereas the PS detected more cases in 2015.

AS is effective in detecting infections in individuals who are less likely to seek medical treatment and individuals with mild illness [12, 13, 15, 16, 24, 25]. Since only individuals with severe dengue (with warning signs) were laboratory confirmed in the PS system, it is not surprising that children and adolescents made up a greater proportion of PS subjects with confirmed dengue illness. Previous literature has shown that the greatest burden of acute dengue infections occurs in children under 10, while the most severe manifestation of dengue is amongst adolescents aged 14 to 20 years who are likely experiencing a second infection [19, 26, 27]. It is possible that adults were underrepresented in the PS because they were less likely to seek clinical care due to mild disease symptoms or preference for alternative strategies to treat symptoms [12]. Our prior experience working in this population and other similar studies would suggest that women, as the primary caregivers, are more likely to take themselves and their children to the doctor [25, 28]. Women may also be at greater risk of dengue exposure if they are home during the day, when the mosquito is actively biting. Adults, and men in particular, may be less likely to seek clinical care due to work demands (i.e., inability to take time off) and their personal preferences. Alternative medicine, over the counter self-medication, and home remedy care are common for dengue treatment in Ecuador [29]. Public health interventions could be tailored to improve dengue case detection in adults, through coordinated action with local pharmacists and alternative medicine practitioners, who often serve as the first point of contact for community members seeking treatment.

During a year with a high burden of dengue (2014), AS was more successful than PS in identifying total cases of dengue illness, as compared to a year with a moderately low dengue burden (2015), when PS performed better. In 2014, AS and PS detected the peak in laboratory confirmed dengue illness around the same time, whereas AS detected a substantially earlier peak in 2015. This suggests that PS is able to accurately detect the initial rise in dengue cases during a high transmission year, but may be prone to underestimating dengue incidence [12, 13, 17]. During a year with lower transmission, PS was slower to detect an initial rise in cases, but was able to accurately capture the burden of illness over the season. Prior studies suggest that dengue cases may go unreported in the PS due to (1) lack of recognition of symptoms by patients or physicians and confusion with other common febrile illnesses, (2) preference for self-treatment of dengue-like symptoms, (3) barriers to accessing healthcare (e.g., cost, transportation), and 4) limited resources of the health system which restrict the ability to confirm a dengue diagnosis [12, 13, 29, 30]. Given the normal year to year variation in dengue transmission, combining information from both systems can provide a more accurate picture of disease burden over time. Other studies have found that complementary AS methods such as syndromic surveillance or sentinel surveillance can lead to earlier detection of outbreaks [14, 17].

The greater effectivity of the PS in detecting dengue illness in 2015 may also be due to the emergence of the first chikungunya epidemic [31], which likely increased health care seeking behavior among symptomatic residents due to public health campaigns resulting in increased awareness. The majority of cases initially recorded in the PS dengue illness (with warning signs) data set were subsequently reclassified with diseases other than dengue following lab confirmation. Despite the high number of cases that were discarded, the PS was still able to identify more total laboratory confirmed dengue infections than the AS. It is also possible that interannual differences between AS and PS were due changes in the predominant virus serotype in circulation (DENV2 in 2014 and DENV1 in 2015) as previously reported [22], thus altering the clinical presentation of dengue infections and prevalence of primary versus secondary dengue infections across age classes.

It is likely that many of possible dengue cases in 2015 were actually chikungunya cases [22], resulting in differences in incidence rates by age groups in the PS dengue illness and possible dengue datasets (Table 1). For example, the increasing incidence of possible dengue in older adults from 2014 (10.21 cases per 10,000) to 2015 (25.26 cases per 10,000) can likely be attributed to cases of dengue that were misdiagnosed and were actually chikungunya. Prior studies from this time period in Machala documented the highest proportion of symptomatic laboratory confirmed chikungunya infections in adults 60–79 years of age, individuals who had been clinically (mis) diagnosed with dengue [22]. Interestingly, the cumulative incidence of the combined PS datasets was similar in both 2014 and 2015 (Table 1), likely reflecting the overall burden of arboviral illness.

Possible dengue cases in the PS system may have been caused by other pathogens. A recent study detected antibodies to spotted fever group rickettsia in 25% of individuals clinically diagnosed with dengue from Machala in 2014–2015 [28]. However, it is unlikely that chikungunya or other mosquito borne diseases were co-circulating in 2014, as reported previously [22]. No Zika cases were detected via AS until 2016 and no malaria cases were reported from 2012 to 2017 [32].

Epidemiological curves during 2014 and 2015 were consistent with previous literature showing peaks in dengue transmission at the end of the hot, rainy season in this region [19]. This timing is likely associated with the presence of open containers and reservoirs of standing water that accumulate in the household patio during the rainy season, which become a habitat for juvenile *Ae. aegypti*. Common larval habitats during the rainy season in Machala includes 55 gallon drums, tires, buckets, wash basins, empty plastic containers and other rubbish [18]. Ambient temperatures during this time of year fall within the optimal temperature range (28.4–29.8 °C) for dengue transmission [22, 33].

The strengths of this study include the ability to compare a research-based AS system to an existing, mandatory PS system in an area with a high burden of arboviral illness. This study also allowed us to compare the performance of these systems over 2 years, before and during the emergence of a novel arbovirus (chikungunya). Since mandatory reporting of dengue fever has been required for some time in Ecuador, laboratory diagnostics were used to confirm dengue illness in a subset of more severe patients in the PS system. Overall, the methods for the collection of AS data were thorough and included similar data to that collected routinely by the MoH. The cluster study design of the AS is relatively efficient compared to more intensive surveillance methods (e.g., cohorts), increasing the likelihood that this could become an operational AS approach for Ministries of Health with limited resources.

There are some limitations in our study, due in part to the observational nature of the design. Although clinical cases without laboratory confirmation were reported as part of PS (possible dengue cases), there was no equivalent category in the AS system. Nonetheless, we were able to compare the demographics of the possible dengue patient group to both the AS and PS dengue illness data sets, highlighting the similarities and differences. Even though index cases (symptomatic individuals who tested positive for dengue) in the AS study were initially identified at one of the MoH sentinel clinics or the hospital, we were unable to identify with certainty those cases that also were reported to the MoH PS system. Consequently, we did not include the AS dengue illness cases in incidence calculations of the total burden of disease in the Machala study area. The reported incidence figures are, therefore, underestimates of the true burden of laboratory confirmed dengue illness in the Machala area. Finally, the incidence proportions reported were calculated based on the population of the Machala area, not the catchment area specific to the clinics or the country level population total. These incidence figures cannot be compared directly to incidence figures calculated either for the catchment area or the country because of the differing denominators employed.

## Conclusions

PS is a useful tool in informing disease trends; however, this approach can lead to mischaracterization of disease burden due to systematic underreporting in certain subpopulations which hinders control efforts and furthers disease transmission [12, 14, 34]. Results from this comparative analysis reveal that AS studies can complement data gathered by PS systems, allowing for an overall improvement in the early detection of cases across a diverse population. This information can be used by health decision makers to improve the allocation of scarce financial and logistical public health resources to prevent outbreaks. A combined surveillance approach at sentinel sites would provide a more accurate estimate of disease incidence and strengthen prevention and control efforts for dengue disease [14, 35].

## Data Availability

The datasets used and/or analyzed during the current study are available from the corresponding author on reasonable request.

## Abbreviations

AS: Active arbovirus research surveillance study
PS: Passive surveillance
MoH: Ministry of Health
Ae. aegypti: *Aedes aegypti*
